# The Importance of Some Symptoms of Uterine Fibroids, with Report of Cases*Read at East Texas Medico-Chirurgical Association, Palestine, Texas, May, 1906.

**Published:** 1906-07

**Authors:** F. L. Barnes

**Affiliations:** Trinity, Texas


For Texas Medical Journal.

### The Importance of Some Symptoms of Uterine Fibroids, with Report of Cases.*

BY F. L. BARNES, M. D., TRINITY, TEXAS.

MR. CHAIRMAN AND GENTLEMEN: It is not my intention at this time to undertake a complete discussion of the symptomatology of uterine fibroids. I merely desire to call your attention to the apparent disproportion which we sometimes find between the size of the tumors and the severity of the symptoms. I also desire to emphasize the fact that these symptoms are probably more often due to adhesions than to the tumor *per se*.

CASE 1. L. P. Was called hurriedly to see this patient May 2, 1901, on account of hemorrhage, pain and retention of urine. Found that the urine had not been passed in twenty-four hours; uterine hemorrhage had lasted eight days, pains were labor-like and severe.

On examination I found a large mass the size of a child's head protruding from vulva. After various unsuccessful efforts to remove this mass, it was finally extracted with obstetric forceps. Uterus was then packed with idoform gauze and she was given stimulants hypodermically. She revived, and on the following day gave this history: Colored, 35 years old, a field hand. She had one child and one miscarriage. Menstrual periods always regular and painless. Found knot in right ovarian region about seven years ago which has continued to enlarge and multiply since. Has sharp pains in back and in tumors with each menstrual period. Lately periods have occasionally been too profuse. Had severe hemorrhage in December lasting three weeks. About normal in

*Read at East Texas Medico-Chirurgical Association, Palestine, Texas, May, 1906.

January and February, but very severe in March and April. Menstrual discharge usually offensive, but not always so. Has severe leucorrhea in interim.

Abdominal examination shows several large tumors entirely filling the abdomen, all connected, but freely movable.

On account of the profuse hemorrhages which recurred when a sub-mucous fibroid would pass, and on account of the discomfort of the greatly distended abdomen, it was decided to do a radical operation. On June 1, 1901, abdomen was opened, and outside of some intestinal adhesions, and some slight difficulty in lifting one large tumor up out of the pelvis, the uterus and tumors were easily removed. Patient made a satisfactory recovery.

Case 2. Bettie G.; colored; about 40; widow. Came under observation about March, 1906. Family history good, and bears no important relationship to this case. She has always had good health, denies rheumatism, syphilis, dropsy, shortness of breath, tuberculosis, etc. Has had no children. Menses appeared at 14; have been regular, painless and of moderate flow.

History of tumors: About two years ago a small, hard tumor was discovered in the left iliac region; has grown rapidly, and other tumors have appeared from time to time. She complains of very slight pelvic pains and pains in the hip joints after working hard.

On examination the abdomen is enormously distended and feels like one large nodular tumor mass. The tumors are movable in mass. Cervix is sound. Uterine canal measures about ten inches.

The symptoms in this case were indeed very slight, and the only reason this woman had for wanting to part with her tumors was because of her very much distended abdomen. She was not operated on because of chronic nephritis.

Case 3. Eliza B.; age 30; colored; widow. General history for self and family good and unimportant.

Special history: Has had no children and no miscarriage. Menses appeared at 14; have been regular, profuse and accompanied by bearing-down pains in left lower abdomen.

Was operated on for some pelvic trouble seven years ago. She thinks it was an abscess.

Present condition: Has had a constant bearing-down pain in left lower abdomen for four years and a constant dull aching pain in lumbar region, also sharp shooting pains in left hip joint, all increased at menstrual period. Has had frequent attacks of cystitis; bowels constipated. About two months ago left hip joint began to pain her much more severely, since which time she has been unable to use the limb.

On examination a small, hard tumor was found to the left and posterior to the lower uterine segment. It was only slightly movable, and being unable to push it up from its incarcerated position, a radical operation was advised for the relief of the various symptoms of pain and invalidism.

At operation it was found that the omentum passed down in front of and around the pelvic organs and was firmly attached to them and to the abdominal and pelvic walls. This was doubly ligated and cut. The surface of the uterus was nodular. The tumor behind and to the left completely blocked those spaces and was firmly fixed by adhesions. The tubes were thickened and the ovaries were cystic. After breaking up the adhesions the uterus, with the tumors were easily removed. This patient made a satisfactory recovery and was entirely cured of all symptoms.

Case 4. Frances H. Colored; age 30; married; one child 8 years old.

Family history: Has no bearing on this case.

Previous history: Enjoyed good health until grown. Menses appeared at 12 and were regular and painless for ten years. Eight years ago the flow became very profuse and painful. Pain appeared one day before flow and continued throughout. Pains have been of crampy variety.

An ovarian cyst was tapped five years ago and about a pint of fluid removed.

Present condition: Menstruation has been present one month with a constant, sharp-cutting pain in lower abdomen. About three weeks ago she noticed for the first time a bulging in the left lower abdomen and could make out a distinct tumor. This has been accompanied most of the time by a sensation of "everything pulling apart" in the pelvis. Lower left quadrant of the abdomen greatly distended by irregular tumor mass, freely movable to the left, motion very limited to the right. Mass occupies left iliac fossa.

At operation it was found that a wide peritoneal band extended from the anterior surface of the tumor mass to the rectum and pelvic wall behind. This band was very tense and evidently gave rise to the "pulling apart" sensation which had been so prominent in her case. There were also numerous other adhesions posterior to the uterus. Both ovaries were cystic, and the right tube was distended with a greenish fluid. After disposing of the adhesions the uterus and tumors were easily removed. After-history of patient entirely satisfactory.

In reviewing these cases I am impressed with the idea that these cases of small fibroids would probably not have given any trouble for a long time had there been no surrounding adhesions, and that the time when they cause the most trouble is just at the time when thev are about to be converted from pelvic into pelvo-abdominal tumors. If they are not bound by adhesions or blocked by irregular formations at this time they will come up into the abdomen like a normally pregnant uterus and the patient will experience considerable relief from pelvic pressure and pain. On the contrary, if they can not make a smooth entry into the abdomen the patient will suffer an exacerbation of all symptoms and probably some new complications.

